# Feasibility, safety, clinical, and laboratory effects of convalescent plasma therapy for patients with Middle East respiratory syndrome coronavirus infection: a study protocol

**DOI:** 10.1186/s40064-015-1490-9

**Published:** 2015-11-19

**Authors:** Yaseen Arabi, Hanan Balkhy, Ali H. Hajeer, Abderrezak Bouchama, Frederick G. Hayden, Awad Al-Omari, Fahad M. Al-Hameed, Yusri Taha, Nahoko Shindo, John Whitehead, Laura Merson, Sameera AlJohani, Khalid Al-Khairy, Gail Carson, Thomas C. Luke, Lisa Hensley, Abdulaziz Al-Dawood, Saad Al-Qahtani, Kayvon Modjarrad, Musharaf Sadat, Gernot Rohde, Catherine Leport, Robert Fowler

**Affiliations:** Intensive Care Department, Respiratory Services, College of Medicine, King Abdullah International Medical Research Center, King Saud bin Abdulaziz University for Health Sciences, King Abdulaziz Medical City, P.O. Box 22490, Riyadh, 11426 Saudi Arabia; Infection Control Department, King Abdullah International Medical Research Center, King Saud bin Abdulaziz University for Health Sciences, King Abdulaziz Medical City, Riyadh, Saudi Arabia; Pathology and Laboratory Department, King Saud bin Abdulaziz University for Health Sciences, King Abdulaziz Medical City, Riyadh, Saudi Arabia; Division of Infectious Diseases and International Health, Department of Medicine, University of Virginia School of Medicine, Charlottesville, VA USA; Critical Care and Infection Control Department, Security Forces Hospital, AlFaisal University, Riyadh, Saudi Arabia; Intensive Care Department, King Saud bin Abdulaziz University for Health Sciences, King Abdulaziz Medical City, Jeddah, Saudi Arabia; Infectious Diseases, Department of Medicine, King Abdulaziz Medical City, Al-Ahsa, Saudi Arabia; Pandemic and Epidemic Diseases Department, World Health Organization, Geneva, Switzerland; Department of Mathematics and Statistics, Lancaster University, Lancaster, UK; Oxford University Clinical Research Unit, 764 Vo Van Kiet Street, Ward 1, District 5, Ho Chi Minh City, Vietnam; University of Oxford Centre for Tropical Medicine (CCVTM), Churchill Hospital Old Road, Headington, Oxford, OX3 7LE UK; Viral and Rickettsial Diseases Department, Naval Medical Research Center, The Henry Jackson Foundation, 6720A Rockledge Drive #100, Bethesda, MD 20817 USA; Integrated Research Facility, National Institute of Allergy and Infectious Diseases, Frederick, MD USA; U.S. Military HIV Research Program, Walter Reed Army Institute of Research, Silver Spring, MD 20910 USA; Intensive Care Department, King Abdulaziz Medical City, Riyadh, Saudi Arabia; Department of Respiratory Medicine, Maastricht University Medical Center, P. Debyelaan 25, 6202AZ Maastricht, The Netherlands; The French Infectious Disease Society, Universite Paris Diderot, Paris, France; Department of Medicine and Institute of Health Policy Management and Evaluation, AMR Infection Control and Publications AIP/PED/HSE/HQ, University of Toronto, Toronto, Canada; Department of Critical Care Medicine and Department of Medicine, Sunnybrook Health Sciences Centre, Toronto, Canada

**Keywords:** Middle east respiratory syndrome coronavirus, MERS-CoV, Viral pneumonia, Intensive care, Convalescent plasma, Serology, Genome, Neutralizing antibodies

## Abstract

As of September 30, 2015, a total of 1589 laboratory-confirmed cases of infection with the Middle East respiratory syndrome coronavirus (MERS-CoV) have been reported to the World Health Organization (WHO). At present there is no effective specific therapy against MERS-CoV. The use of convalescent plasma (CP) has been suggested as a potential therapy based on existing evidence from other viral infections. We aim to study the feasibility of CP therapy as well as its safety and clinical and laboratory effects in critically ill patients with MERS-CoV infection. We will also examine the pharmacokinetics of the MERS-CoV antibody response and viral load over the course of MERS-CoV infection. This study will inform a future randomized controlled trial that will examine the efficacy of CP therapy for MERS-CoV infection. In the CP collection phase, potential donors will be tested by the enzyme linked immunosorbent assay (ELISA) and the indirect fluorescent antibody (IFA) techniques for the presence of anti-MERS-CoV antibodies. Subjects with anti-MERS-CoV IFA titer of ≥1:160 and no clinical or laboratory evidence of MERS-CoV infection will be screened for eligibility for plasma donation according to standard donation criteria. In the CP therapy phase, 20 consecutive critically ill patients admitted to intensive care unit with laboratory-confirmed MERS-CoV infection will be enrolled and each will receive 2 units of CP. Post enrollment, patients will be followed for clinical and laboratory outcomes that include anti-MERS-CoV antibodies and viral load. This protocol was developed collaboratively by King Abdullah International Medical Research Center (KAIMRC), Gulf Cooperation Council (GCC) Infection Control Center Group and the World Health Organization—International Severe Acute Respiratory and Emerging Infection Consortium (ISARIC-WHO) MERS-CoV Working Group. It was approved in June 2014 by the Ministry of the National Guard Health Affairs Institutional Review Board (IRB). A data safety monitoring board (DSMB) was formulated. The study is registered at http://www.clinicaltrials.gov (NCT02190799).

## Background

The Middle East respiratory syndrome coronavirus (MERS-CoV) was initially identified in September 2012 from samples obtained from a Saudi Arabian patient who developed severe acute respiratory infection and subsequent acute renal failure leading to death (Zaki et al. 2012). As of September 30, 2015, a total of 1589 cases have been identified with 567 related deaths (World Health Orgnization 2015). To date, there is no specific treatment of proven effect for MERS-CoV infection. Public Health England and the International Severe Acute Respiratory and Emerging Infection Consortium (ISARIC) have published a decision support tool for clinicians managing cases of MERS-CoV infection. The document suggests that current evidence is strongest for testing convalescent plasma (CP) or other therapeutics which contain neutralizing antibodies (such as hyperimmune immunoglobulin) for treatment of serious MERS-CoV illness (Public Health England 2015). Prior experience in SARS and severe influenza suggest that CP may be considered for patients who are deteriorating (despite other specific and supportive therapy) and in whom the virus remains detectable (Hung et al. 2011; Luke et al. 2006; Cheng et al. 2005; Kong and Zhou 2006; Yeh et al. 2005). A recent systematic review of 32 reports from SARS and severe influenza concluded that CP therapy appears safe and may reduce mortality, especially if administered early in the illness (Mair-Jenkins et al. 2015). An exploratory post hoc meta-analysis showed a statistically significant reduction in the pooled odds of mortality following treatment compared to placebo or no therapy (odds ratio 0.25; 95 % confidence interval 0.14–0.45; I^2^ = 0 %) (Mair-Jenkins et al. 2015). Citing case series, the authors commented that (1) patients with severe presentations appeared to demonstrate temporal clinical improvements after treatment with CP and (2) administration as early as possible in the diseases course appears to be associated with greatest potential clinical effect. One randomized clinical trial (RCT) in critically ill influenza A (H1N1pdm09)-infected patients found a survival benefit when hyperimmune globulin was administered within 5 days of symptom onset (Hung et al. 2013).

However, there are no data at present to support the efficacy of CP treatment in MERS-CoV infection; therefore, it has been recommended to administer CP only in the context of a clinical trial. While an RCT will be required to evaluate effectiveness, evaluating effectiveness on clinical endpoints such as mortality will likely require several hundred to several thousand seriously ill MERS-CoV patients in order to achieve sufficient statistical power, anticipating reasonable potential effect sizes. Additionally, CP from different MERS-CoV survivors will likely contain differing levels of neutralizing anti-MERS-CoV antibodies. Since seriously ill MERS-CoV-infected patients may have detectable viral RNA in various locations that can be sampled (for example lower respiratory tract secretions) for prolonged periods, it might be possible to first determine the relationship between neutralizing antibody dose and antiviral effects on clinical and laboratory features in a small open-label study. This information would be very helpful to design of an RCT and in determining the most appropriate neutralizing antibody dose, or dosing range for the study. This may also inform dose selection for follow-on anti-MERS-CoV antibody preparations currently in pre-clinical development (for example, neutralizing human monoclonal antibodies, polyclonal human neutralizing immunoglobulin derived from transchromosomic cattle (personal communication, Thomas C. Luke).

Therefore, we plan to conduct a 2-phase study. In the first phase (CP collection phase), we will explore the feasibility of collection of CP from donors who have significant titers of anti-MERS-CoV antibodies. In the second phase, patients with MERS-CoV infection will be treated with CP. If the protocol is feasible, safe, and associated with temporal changes in viral load and illness, this pilot study will inform a larger concealed intervention, placebo-controlled RCT that is powered to evaluate efficacy of CP on relevant clinical outcomes.

## Methods

### Study population

#### CP collection phase

The inclusion criteria for screening potential CP donors include individuals from the following cohorts: (1) healthcare workers (HCWs) who had documented exposure to MERS-CoV, (2) recovering patients from confirmed or suspected MERS-CoV infection, (3) household contacts of known MERS-CoV infected patients and (4) other subjects who are willing to donate plasma. Females with prior pregnancy will not be included for donation.

#### CP therapy phase

We will screen consecutive critically ill patients admitted to the intensive care unit or other areas of the hospital where critically ill patients receive care for the following criteria:

Inclusion criteria

1. Critical illness as defined by one or more of the following: admission to an ICU; current receipt of mechanical invasive or non-invasive ventilation; partial pressure of oxygen to fraction of inspired oxygen ratio (PaO2:FiO2) of <300 mmHg; current receipt of intravenous vasoactive medications to maintain mean arterial pressure >65 mmHg; new-onset (since development of MERS-CoV symptoms) receipt of renal replacement therapy or extra-corporeal life support.
2. Laboratory-confirmed MERS-CoV infection (by real-time reverse-transcription polymerase chain reaction rRT-PCR).
3. Age of more than or equal to 14 years.

Exclusion criteria

1. Symptomatic illness exceeding two weeks (14 days) at time of enrollment.
2. Negative rRT-PCR from respiratory secretions or blood within 48 h prior to assessment of eligibility.
3. History of allergic reaction to blood or plasma products (as judged by the investigator).
4. Known IgA deficiency.
5. Medical conditions in which receipt of 500 mL intravascular volume may be detrimental to the patient (e.g., actively decompensated congestive heart failure).

#### Informed consent

The research coordinator and/or physician investigator will explain the objectives of this study and its potential risks and benefits to the donor or patient (or to his/her surrogate decision maker) and will obtain the following consent forms in and as appropriate:

CP collection phase

1. Consent for MERS-CoV serologic testing and MERS-CoV RT-PCR for donors.
2. Consent for CP donation for those who have elevated anti-MERS-CoV titers as described below.

CP therapy phase

1. Consent for enrollment in the CP therapy phase.
2. Consent for enrollment in the observational study where NO intervention will be received, but participants will still have blood (and possibly respiratory) samples taken—for participants not receiving the intervention.

### Study procedures

#### For the CP collection phase

1. Eligible candidates for CP donation (as per the inclusion and exclusion criteria above) will be approached to have their blood tested for anti-MERS-CoV serology (see laboratory methods). Subjects who are seropositive will be screened subsequently for MERS-CoV rRT-PCR to exclude active infection.
2. Subjects with anti-MERS-CoV-specific titer ≥1:160 and no clinical (not requiring medical support for respiratory or other organ function) or laboratory (rRT-PCR negative) evidence of MERS-CoV infection will be screened for eligibility for plasma donation according to the standard criteria in accordance with the WHO Guidelines Assessing Donor Suitability for Blood Donation (The World Health Organization 2015).
3. Those who meet the plasma donation criteria will be invited for donation according to the WHO Blood Regulators Network (BRN) Position Paper on Collection and use of convalescent plasma or serum as an element in Middle East respiratory syndrome coronavirus response (WHO Blood Regulators Network (BRN) 2015). Plasma may be collected by apheresis as frequently as twice every month, as appropriate for the individual donor. Collection will be performed by trained blood bank staff operating under the standard operating procedures in certified facilities. The collected frozen plasma will be stored in the blood bank after being tested for serology of hepatitis B and C viruses (HBV and HCV), human immunodeficiency virus (HIV), malaria, syphilis and human T-lymphotropic virus (HTLV) types I and II and nucleic acid testing (NAT) for HBV, HCV and HIV according to international guidelines.

#### For the CP therapy phase

1. Critically ill MERS-CoV patients who meet the above patient eligibility criteria will be approached for consent.
2. Patients will have their blood type determined. CP must be ABO compatible with the recipient’s blood type.
3. The trial intervention include the administration of 2 units of CP. Each unit of plasma will be given over 2 h with an interval of 1 h between the two units. Plasma transfusion will be done in accordance with the standard policies for administration of blood products.

#### Co-interventions

The clinical team will have full, independent control of patient management and as such, management other than CP therapy will not be influenced by the intervention or study team. Co-interventions, including corticosteroids, ribavirin, intravenous immunoglobulin and interferon, will be documented on the study case report forms.

#### Co-enrollment

Co-enrollment in another study is permissible as long as the enrollment in the other study would not be at moderate to high risk of biologically or analytically confounding the results of this study, as judged by the study management committee and as per the published guidelines.

#### Frequency and duration of follow-up

Clinical and laboratory data will be collected at baseline, 30 min after first dose, 30 min after second dose, study days 1, 3, 5, 7, 14, and 28.

### Outcome measures

#### CP collection phase

We will explore the feasibility of the study intervention, as measured by ability to screen potential plasma donors, and derive sufficient plasma to enrol 20 patients in a 12 months period. We will also qualitatively describe logistical challenges experienced through the conduct of this study, including ethical, administrative and regulatory challenges.

#### CP therapy phase

1. We will establish safety of the study intervention, as measured by number of serious adverse events related to study intervention (adverse events include development of complications of intravascular volume overload and clinical pulmonary edema by temporally related-shortness of breath, chest radiograph findings and change in oxygenation requirements; development of transfusion-related acute lung injury (TRALI) or substantial allergy or anaphylaxis). These serious events will be adjudicated by a committee of 3 investigators.
2. *Clinical Outcomes* We will measure (1) sequential organ failure assessment (SOFA) scores on study days 1, 3, 5, 7, 14, and 28 (2) requirement for organ support (oxygen and ventilation; dialysis; vasopressors) after enrollment; (3) length of stay in ICU defined as the number of calendar days between admission and final discharge from ICU for the same ICU admission of enrollment; and duration of mechanical ventilation, defined as the number of calendar days between start and final liberation from mechanical ventilation for the same ICU admission of enrollment and hospital length of stay as defined as the number of calendar days between admission to hospital and final discharge from hospital for the same hospital admission; and (4) vital outcome (mortality) in ICU, hospital and at 28 days.
3. Other clinical outcomes include “ICU-free days”, defined as the number of days that patients are not in ICU in the first 28 days after enrollment. Patients who die within 28 days will be counted separately, and not categorised by ICU-free days. Similarly, “ventilator-free days” is defined as the number of days that patients do not receive mechanical ventilation in the first 28 days after enrollment. “Renal replacement therapy-free days” and “vasopressor-free days” are defined in a similar way. Serial chest radiograph findings, as obtained by the clinical team will also be recording as per case report form, graded as unilateral or bilateral infiltrates, in 1–4 quadrants.
4. *Laboratory Outcomes* We will measure the following laboratory outcomes:
  - The serum level of anti-MERS-CoV antibodies before and after administration of CP.
  - MERS-CoV viral load (the primary laboratory outcome is viral clearance from all sampled sites by day 3 after administration of CP).

### Laboratory procedures

1. **Measuring anti-MERS-CoV antibodies level in donor and participant serum**
  MERS-CoV antibodies will be tested first by the enzyme linked immunosorbent assay (ELISA) as a screening test (Drosten et al. 2014; Müller et al. 2015) according to manufacturer’s instructions (Euroimmun AG, Lübeck, Germany). Results will be reported as the optic density (OD) ratio, which is calculated as the OD value of the patient’s sample divided by the calibrator OD value. We will use the cut-off values recommended by the manufacturer: a ratio of <0.8 is considered negative, >0.8 and <1.1 borderline and a ratio of >1.1 is considered positive.
  Confirmation will be done by the Indirect Fluorescent Antibody (IFA, Euroimmun AG, Lübeck, Germany) according to manufacturer’s instructions. Samples with ≥1:10 will be considered reactive according to the manufacturer’s instructions, subjects will be considered candidate for plasma donation if they have titers of ≥1:160; which is a similar threshold to what has been used in a convalescent plasma trial for H1N1 influenza (Hung et al. 2011).

### Administrative and ethical aspects

The primary coordinating study center is the Intensive Care Department at King Saud bin Abdulaziz University for Health Sciences (KSAUHS) in Riyadh, Saudi Arabia. The study will be conducted in accordance with the ethical principles of the Declaration of Helsinki and the International Conference on Harmonization-Good Clinical Practice (ICH-GCP) guidelines.

Several measures will be taken to ensure optimal compliance with the study protocols. Before launching the study, ICU physicians and nurses will attend the training sessions with special emphasis on any adverse events noted during the intervention. The Steering Committee, led by the principal investigator, will be responsible for overseeing the conduct of the trial, for upholding or modifying study procedures as needed, addressing challenges with protocol implementation, formulating the analysis plan, reviewing and interpreting the data and preparing the manuscript. The study also has an independent data safety monitoring board (DSMB) which is responsible for reviewing reports submitted to the regarding safety of study patients, protocol adherence and may making recommendations to continue or terminate the study based on safety analysis results. The DSMB, composed of 5 members (who are named at the end of this document) will meet at the beginning of phase II of the study followed by 6-monthly or as needed.

#### Safety measures

In the event of an acute transfusion reaction, the transfusion will be stopped immediately and must be reported to the blood bank the principal investigator immediately as well as to the study management committee. All the serious adverse events (SAE) adjudicated as related to the study intervention will be reported to the Institutional Research Ethics Board and the DSMB.

### Statistical and analytical plan

#### Sample size calculation

This is an exploratory study, aimed at rectifying the current lack of information on the use of CP to treat MERS-CoV infection. Due to the exploratory nature of this study and the paucity of sequential data on viral RNA levels in respiratory tract and blood samples from MERS-CoV-infected patients, and on their clinical progress, the sample size is fixed at 20, which is a realistic target for a study of 12 months duration. The sample size of 20 is sufficient to reach a conclusion that the 28-day survival rate significantly exceeds 60 % (p = 0.032, 2-sided) if 17 or more patients survive for the 28 days of follow-up. This would represent promising evidence to motivate a full-scale comparative clinical trial.

### Statistical analyses

#### Analysis of viral load data

1. Serial MERS-CoV viral load measurements will be displayed as box and whisker plots for the 20 treated patients against time.
2. The probability of a patient having an undetectable viral load from all sampled sites by day 3 after administration of therapy will be estimated by the proportion of the 20 treated patients for whom this occurs. An exact, conservative, two-sided confidence interval for this probability will be calculated using the method of Clopper and Pearson (1934).
3. The relationship between log viral load at day 3 and the neutralizing antibody dose received will be characterised by fitting a regression model to the data from the 20 treated patients. The log viral load at baseline will be included in this model.
4. The relationship between the probability of a patient having an undetectable viral load by day 3 and the neutralizing antibody dose received will be characterised by fitting a log-logistic regression model to the data from the 20 treated patients. The log viral load at baseline will be included in this model.

#### Analysis of clinical data

1. The SOFA score and indicators of whether the patient requires organ support via oxygen and ventilation, dialysis or vasopressors will be plotted against time.
2. The relationships between the SOFA score at day 3 and the neutralizing antibody dose received, and between receipt of any type of organ support during the 28 days of observation and the neutralizing antibody dose received, will be characterised by fitting a logistic regression model to the data from the 20 treated patients. The log viral load at baseline will be included in these models.
3. The vital status (alive or dead) of each patient will be recorded for all days 0–28. The proportion alive will be plotted against time.
4. The relationship between the hazard of death and the neutralizing antibody dose received will be characterised by fitting a Cox proportional hazards regression model to the data from the 20 treated patients. The log viral load at baseline will be included in this model.
5. The probability of a patient dying on or before 28 days will be estimated by the proportion of the 20 treated patients for whom this occurs. An exact, conservative, two-sided confidence interval for this probability will be calculated using the method of Clopper and Pearson (1934).
6. The time from infection/exposure and sample collection in days, duration from infection/exposure to CP therapy, length of stay in ICU; the number of ICU-free days; the duration of mechanical ventilation; the numbers of ventilator-free days, of renal replacement therapy-free days, and of vasopressor-free days; and the length of stay in hospital will be presented as histograms, and suitable summary statistics will be computed.

### Stratified analyses

We will conduct exploratory stratified analyes based on (1) the time between symptom onset and CP therapy initiation, (2) comorbidities, (3) co-intervention; and (4) baseline severity (SOFA scores) at treatment initiation.

The SAS System for Windows version 9.3 (SAS Institute, Inc., Cary, North Carolina) and R will be used for all analyses.

### Discussion and current status

This protocol was developed collaboratively by King Abdullah International Medical Research Center (KAIMRC), Gulf Cooperation Council (GCC) Infection Control Center Group and the World Health Organization—International Severe Acute Respiratory and Emerging Infection Consortium (ISARIC-WHO) MERS-CoV Working Group. It was approved by the Ministry of the National Guard Health Affairs Institutional Review Board (IRB) (approval number IRBC/13/244, 5th June 18, 2014) and has been registered at clinicaltrials.gov (NCT02190799).

If proven effective, CP therapy is an attractive therapeutic option for MERS-CoV infection. Besides the biologic plausibility of this therapy, it is easy to obtain and administer, relatively inexpensive, and is likely to be acceptable to patients and treating teams. Side effects are unlikely to differ from those of transfusion of any other fresh frozen plasma. We believe this study protocol sets the stage to a large efficacy trial.

The strengths and weaknesses of the study protocol should be noted. In the CP collection phase subjects will be enrolled from 4 different cohorts, in order to explore all potential donors. It is unknown, at this point, which subjects are likely to have high antibody titers and therefore be CP donors. We are hoping that this feasibility study will help identifying a group of superdonors who have very high titers. By identifying the characteristics of such individuals, a more focused approach for donation can be followed. The CP therapy phase is not designed to establish efficacy; such objective requires an adequately powered randomized controlled trial. However, we believe performing this feasibility study is an essential step to examine the safety, clinical and laboratory effects and the pharmacokinetics of the MERS-CoV antibody response. The study involves giving critically ill patients this therapy in a controlled monitored setting. However, a recent systematic review suggested that early treatment with CP is likely to be more effective than late treatment (Mair-Jenkins et al. 2015). Therefore, if the feasibility study shows that CP is safe and feasible, the next step should be a randomized controlled trial that is sufficiently powered to detect effect on mortality and enrolls patients early in the course of the disease.

## Conclusions

Our study is anticipated to provide information about the feasibility of collecting convalescent plasma in large quantities for therapeutic use in a large numbers of MERS-CoV patients. The data is anticipated to inform about the relation between the antibody titers in the CP and viral clearance and other laboratory and clinical endpoints. This data will be critical in planning a larger RCT to examine the efficacy of CP on patients with MERS-CoV infection.

## Abbreviations

MERS-CoV: Middle East respiratory syndrome coronavirus
ICU: Intensive care unit
PCR: Polymerase chain reaction
ISARIC: International Severe Acute Respiratory and Emerging Infection Consortium
SARS: Severe acute respiratory syndrome
CP: Convalescent plasma
HCW: Health care workers
WHO: World Health Organization
ELISA: Enzyme linked immunosorbent assay
IFA: Indirect fluorescent antibody
